# Virtual robotic telepresence early childhood mental health consultation to childcare centers in the aftermath of COVID-19: training approaches and perceived acceptability and usefulness

**DOI:** 10.3389/fpsyg.2024.1339230

**Published:** 2024-06-06

**Authors:** Jason F. Jent, Sara M. St. George, Yaray Agosto, William A. Rothenberg, Elizabeth Howe, Carolina Velasquez, Elana Mansoor, Emperatriz G. Garcia, Rebecca J. Bulotsky-Shearer, Ruby Natale

**Affiliations:** ^1^Leonard M. Miller School of Medicine, University of Miami, Miami, FL, United States; ^2^Department of Psychology, University of Miami, Coral Gables, FL, United States

**Keywords:** telepresence robot, teleconsultation, training, early childhood consultation, childcare, technology acceptability, COVID-19 virtual robotic telepresence consultation

## Abstract

**Introduction:**

Childcare center closures during COVID-19 impacted education for approximately 40 million children nationwide. Unfortunately, COVID-19 restrictions significantly limited the extent that outside personnel could provide in-person support to educators, resulting in the need for innovative approaches to meet childcare centers’ needs. A virtual robotic telepresence approach was applied to early childhood consultation models to promote child resilience while mitigating COVID-19 risks. The goal of this study was to examine how training influenced consultants’ and childcare staff uptake of the virtual robotic telepresence consultation approach and their acceptance of this technology.

**Methods:**

Ten early childhood consultants received multimedia/simulation training and weekly communities of practice related to virtual telepresence robotic consultation. Telepresence robotic consultation equipment was deployed to 16 childcare centers in a diverse multilingual metropolitan area as a part of a larger randomized controlled trial. Consultants trained childcare staff (14 center directors and 58 teachers) on how to receive virtual telepresence robotic consultation. Demographic information and measures of technology acceptability and uptake were collected from childcare staff and consultants. A mixed methods approach was used including multilevel modeling and focus groups to examine consultation uptake, acceptability, barriers, and facilitators of virtual telepresence robotic consultation implementation.

**Results:**

Consultants and childcare staff generally perceived the virtual telepresence consultation approach to be useful and easy to use. Consultant perceptions of the acceptability of technology did not change over time. Childcare staff, center, and consultant factors impacted the uptake of the virtual robotic telepresence consultation approach and childcare staff acceptance of the technology. Focus groups revealed that consultants believed that additional hands-on training with childcare staff would have benefited implementation and expressed a desire for a hybrid approach for consultation.

**Discussion:**

Perceptions of telepresence robotic consultation acceptability are discussed, including future recommendations for training.

## 1 Introduction

The closure of childcare centers in 2020 due to COVID-19 meant that 40 million young children in the U.S. missed out on early childhood education during critical toddler and preschool years (UNICEF, 2023). The unprecedented disruptions during the COVID-19 pandemic presented challenges to an already overburdened childcare system (Kalluri et al., 2021). A key challenge for childcare centers was reestablishing and strengthening the physical and psychological wellbeing of children so essential for healthy early child development. For childcare centers to meet this basic need, teachers/staff needed support, resources, and consultation to provide them with strategies on how to support healthy child development and behavior (Natale et al., 2023). Prior to COVID-19, support to childcare centers was typically provided through in-person support and early childhood mental health consultation models (Gilliam et al., 2016; Natale et al., 2020). However, during the pandemic, many childcare centers restricted outside visitors into their centers to reduce risk of transmission of COVID-19. This resulted in a need for a mechanism to provide support to childcare center directors and teachers remotely. The utilization of a virtual telepresence robotic consultation approach to provide support to childcare centers presented a unique opportunity to be virtually present within the classroom while preventing risk of virus transmission. Virtual telepresence robotic consultation allowed providers to remotely control a stationary robot with a videoconferencing capable tablet by moving the camera field of view and microphone so they can see and hear by following interactions throughout a defined space (Fischer et al., 2018). However, a key challenge to effectively implementing telepresence robot consultations for childcare centers was how to effectively train consultants and childcare staff to promote uptake and acceptability of the approach (Proctor et al., 2011). Therefore, the purpose of this study was to evaluate how a training and community of practice approach impacted consultants and childcare staff’s uptake of the virtual telepresence robotic consultation approach and perceived acceptability within low income, multilingual, historically marginalized communities.

### 1.1 Early childhood mental health consultation

Early childhood mental health consultation models such as the Georgetown Model of Early Childhood Mental Health Consultation (ECMHC) aim to build the capacity of early childhood educators to promote young children’s social–emotional development and effectively address challenging behaviors (Upshur et al., 2009; Heller et al., 2011). Within ECHMHC models, mental health consultants work collaboratively with early childhood programs, such as childcare centers, to enhance the skills and knowledge of educators, foster positive teacher-child interactions, and create supportive classroom environments that nurture children’s social–emotional well-being (Gilliam et al., 2016; Conners-Burrow et al., 2017). The Georgetown Model operates at multiple levels, including programmatic consultation to improve overall program quality, classroom-level consultation to enhance teacher practices and classroom climate, and child-specific consultation to address individual children’s needs (Upshur et al., 2009; Heller et al., 2011). Mental health consultants use a strengths-based, collaborative approach to build relationships with early childhood educators and provide ongoing support, guidance, and professional development (Raver et al., 2008; Duran et al., 2010). The model has demonstrated positive outcomes, such as reduced teacher stress, improved teacher-child interactions, and decreased challenging behaviors in young children (Heller et al., 2011; Gilliam et al., 2016; Conners-Burrow et al., 2017).

Community-specific adaptations of the Georgetown model (i.e., Jump Start) for low-income and predominantly historically marginalized communities have also been implemented. Jump Start is a multi-level model of early childhood mental health consultation in which child-, classroom-, and program-level consultations are provided. Jump Start implementation has been found to have positive effects on child-level (i.e., social–emotional competence) and teacher-level outcomes (i.e., increased use of effective teaching practices; Natale et al., 2020). Jump Start like many other health and behavioral health models had to shift its implementation modality because of COVID-19, due to local and program-specific guidelines and visitor restrictions. Therefore, Jump Start was adapted into a virtual consultation model (Jump Start + COVID Support) to provide tailored COVID-19 specific supports to childcare centers.

The Jump Start + COVID Support (JS + CS) model, is a community-based intervention that aims to support childcare centers in promoting young children’s social–emotional development and resilience, particularly in the context of the COVID-19 pandemic (Natale et al., 2023). JS + CS utilizes mental health consultants to deliver a multimedia toolkit and provide virtual consultation to childcare center directors, teachers, and families (Natale et al., 2020, 2023). The JS + CS program provides consultation on four pillars consistent with CDC COVID-19 guidelines and addresses likely sequalae that could emerge because of COVID-19: (1) safety (e.g., brief cartoon videos about hand washing, wearing masks, and social distancing), (2) communication (e.g., setting up mobile platforms for ongoing communications between families and their teachers regarding their child and COVID-related updates for the center), (3) self-care (e.g., engagement in outdoor exercise, daily mindfulness practices), and (4) Trauma-Informed Behavior Support (e.g., consideration of child’s exposure to trauma or COVID-related disruptions or losses that may impact behavioral and emotional presentation within the classroom; Natale et al., 2023). The four pillars were consistent with the pillars of the original Jump Start model but were tailored to provide additional support and guidance related to COVID-19. To provide the adaption and implementation of JS + CS, a training and community of practice model was developed in hopes of supporting effective implementation.

### 1.2 Virtual consultation within educational settings

Historically, educational settings (e.g., schools, childcare centers) have preferred consultants travel to physical locations and meet in-person with staff to provide consultations. However, COVID-19 restricted outside visitor access to physical locations (Centers for Disease Control and Prevention, 2023). Therefore, consultation programs needed to pivot to be able to continue to provide support to educational settings. Virtual consultation (via video conferencing software) provided an access point for consultation when on-site consultations were not allowed (Natale et al., 2023).

In fact, prior to COVID-19, virtual consultation approaches have been utilized within educational settings to reduce consultant and center barriers to services due to distance and/or commute times (Alnemary et al., 2015; Schultz et al., 2017; Bloomfield et al., 2018). However, consultant perceptions of the consultees’ general technology proficiency (i.e., unfamiliar), and severity of school-based problems (i.e., more severe child behaviors) negatively impacted their preference for utilizing teleconsultation versus in-person consultation approaches (Schultz et al., 2017). Despite these hesitations, consultants and educators have generally viewed a teleconsultation approach as an acceptable intervention approach (Bice-Urbach and Kratochwill, 2016; Fischer et al., 2016; Ihorn and Arora, 2018). It is possible that advances in virtual technology may help increase uptake of virtual consultation approaches.

Specifically, telepresence robots have emerged as tools to increase the extent that someone can feel virtually present within virtual consultations. The goal of telepresence robotic consultations is to recreate in-person interactions as much as possible. Therefore, a virtual telepresence robotic consultation approach requires specific technology to create a sense of telepresence, which gives the user a sense of feeling and control as if they were physically immersed in that remote setting (Minsky, 1980). To be virtually telepresent within a classroom, videoconferencing equipment and software is needed to ensure the remote consultant can adequately see and hear teacher-child interactions within the classroom. Given that teacher-child interactions are fluid, the utilization of a telepresence robot may be helpful as consultants can remotely operate a robot to pan and tilt a mobile device connected to video conferencing software so the consultant can follow the interactions throughout the classroom.

Initial examinations of telepresence robotic consultation approaches have proven effective in hard-to-access schools (Fischer et al., 2019), but research assessing their use in childcare settings is limited. Further, technology-based interventions have also historically been viewed as less acceptable for low-income and minority populations due to failures in tailoring interventions to the literacy, language, and available technology for the target population (Porter and Donthu, 2006; Katz and Gonzalez, 2016; Anderson-Lewis et al., 2018). Additionally, there is mixed evidence regarding the acceptability and utilization of new technology based on age of the user (Berkowsky et al., 2017; Kuerbis et al., 2017; Calvo-Porral et al., 2019). Therefore, evaluations of virtual telepresence robotic consultation training approaches for consultants and childcare staff within low-income and predominantly historically marginalized communities are needed to better understand what factors impact uptake and acceptability of this consultation approach.

### 1.3 Current study

This study took place from November 2022 to September 2023 and is part of a larger cluster randomized controlled trial examining the effectiveness of a virtual robotic early childhood mental health consultation approach on childcare staff and child outcomes in response to COVID-19 (Natale et al., 2023). Within this trial, childcare centers were either randomly assigned to Jump Start + COVID Support or an attention-control early childhood consultation model, Healthy Caregivers, Healthy Children (HC2; Natale et al., 2021). Both interventions were adapted to be administered via a virtual robotic early childhood mental health consultation. Therefore, both the intervention and control groups were included in the current study to better understand telepresence robotic virtual consultation implementation. The larger cluster randomized controlled trial utilizes a Reach (e.g., How do we reach and support childcare staff during COVID-19?), Effectiveness (e.g., What is the effectiveness of Jump Start + COVID Support in improving child, teacher, and program outcomes?), Adoption (e.g., What is the uptake of the consultation approach? What are the barriers and facilitators of virtual telepresence robotic consultation approach), Implementation (e.g., To what degree were consultations delivered virtually as intended?), and Maintenance (e.g., Are the effects of JS + CS maintained long-term after the initial intervention) evaluation framework. The Reach, Effectiveness, Adoption, Implementation, Maintenance (RE-AIM) evaluation framework is designed to help understand factors impacting uptake, successful execution, and sustainability of interventions and has been utilized to analyze childcare center-based interventions (Glasgow et al., 2019). The Reach, Effectiveness, Adoption, Implementation, Maintenance (RE-AIM) framework was initially designed to measure progress in implementation of public health programs, to produce a more balanced approach to internal and external validity, and to address key issues important for dissemination and generalization (Glasgow et al., 1999). The RE-AIM framework has been applied to community-based multilevel interventions (Glasgow et al., 2011) and used to reduce health disparities (Glasgow et al., 2013), which is ideal for the diversity of our community. Within the current study, we were particularly interested in examining Adoption and Implementation of the RE-AIM framework as it related to consultant and childcare staff utilization and perceptions of the virtual robotic telepresence consultation approach.

To evaluate the impact of the novel virtual robotic telepresence consultation approach on consultant and childcare staff uptake and perceptions of this model (Adoption and Implementation), we used a convergent parallel mixed methods design, or one in which quantitative and qualitative data are collected and analyzed simultaneously to evaluate the virtual robotic telepresence consultation approach using a RE-AIM framework (Creswell and Clark, 2017). The training and consultation approach for uptake of using a virtual robotic telepresence consultation in the JS + CS and the HC2 programs were examined using the RE-AIM framework in the following ways: Adoption: Factors influencing adoption, such as barriers and facilitators to uptake of the virtual telepresence robotic consultation approach, were explored through focus groups with consultants. In addition, we predicted that through initial training and ongoing communities of practice, consultant perceptions of virtual telepresence robotic consultation usefulness, ease of use, and predicted future usage would improve over time. To explore what influenced adoption, we also examined how childcare staff, childcare center, and consultant characteristics impacted childcare center staff perceptions of the usefulness, ease of use, and predicted future use of a virtual robotic telepresence consultation approach. Implementation: The fidelity of the consultation being delivered via telepresence robots was evaluated by examining the percentage of consults that were completed virtually relative to in-person, and hybrid modalities (e.g., delivering the intervention virtually, but while onsite at a childcare center). We examined the extent childcare staff, childcare center, and consultant factors influenced the percentage of total consultations conducted via virtual robotic telepresence consultations to better understand what impacted fidelity to the virtual robotic telepresence consultation approach. The findings of this study will be used to identify mechanisms that can promote increased adoption and implementation of the virtual robotic telepresence consultation approach within childcare centers in subsequent trial cohorts, including adaptations to the intervention.

Evaluation of virtual telepresence robotic consultation training approaches and subsequent implementation is vital to understanding how to best support educators during public health crises. The use of stationary telepresence robots provides an affordable teleconsultation mechanism and approach that can be adapted to diverse settings well beyond just childcare centers so understanding their use can provide helpful information to other disciplines providing virtual robotic telepresence consultation.

## 2 Materials and methods

### 2.1 Participants

Sixteen early childcare centers (*n* = 14 center directors, *n* = 58 teachers) across two major metropolitan areas within the southeastern United States were included in Cohort 1 of this study. Centers inclusion criteria were as follows: (1) have ≥50 children (≥30 of whom are 18 month-3 years old); (2) be located in the low-income census tract, with at least 50% of families receiving childcare subsidy; (3) serve at least 60% Hispanic or 60% Non-Hispanic Black families; (4) have directors and teachers who agree to participate; and (5) were not previously enrolled in an early childhood mental health consultation program. Each childcare center and all staff within the center were randomly assigned to receive one of two early childhood consultation models via virtual robotic telepresence consultation. Childcare center directors and teachers were included in the study if they completed a post measure of technology acceptability of the virtual robotic telepresence consultation approach. See Table 1 for childcare staff demographics. Demographic information and pre-post measures were collected via paper surveys and through an online database management system, Research Electronic Data Capture (REDCap; Harris et al., 2009) based on participant preferences. Institutional Review Board (IRB) approval was obtained from the university and all participants who agreed to be in the study signed an informed consent. All study procedures were conducted in accordance with the ethical standards of the IRB.

**Table 1:** Childcare center staff demographics.

Childcare staff (directors and teachers)	
Age in years: *M (SD)*	48.8 (12.3)
	Percentage
Race	
White	88.4%
Black	4.3%
Multiracial	1.4%
Other	2.9%
Ethnicity	
White Hispanic	91.3%
White Non-Hispanic	1.4%
Black Non-Hispanic Black	2.9%
Haitian	1.4%
Gender-Female	100.0%
Primary language	
English	9.0%
Spanish	89.6%
Creole	1.4%
Education	
Associate’s Degree	14.5%
Bachelor’s Degree	20.3%
Master’s Degree	65.2%
Intervention Group	
JS + CS	52.2%
HC2	47.8%
Participant role	
Director or Assistant Director	19.1%
Teacher	80.9%

*N* = 72, 4 participants had missing demographic information; JS + CS, JumpStart + COVID Support; HC2, Healthy Caregivers-Healthy Children.

#### 2.1.1 Consultants

Across the two intervention groups, consultants (*N* = 10) were on average 26 years old (*SD* = 11.89 years). Consultants were similar to childcare center staff as it relates to race and ethnicity (90% White Hispanic, 10% Black Haitian). Consultants had an average of 3.05 years (*SD* = 4.41 years) of early childhood consultation experience and ranged in education level (10% Associate’s Degree, 40% Bachelor’s Degree, 60% Master’s Degree). 100% of consultants were bilingual (90% English/Spanish, 10% English/Creole). Consultants generally reflected the racial, ethnic, and linguistic background of the childcare staff they served. In addition, there were no sociodemographic differences between consultants across intervention groups in terms of consultant age (*F* = 3.46, *p* = 0.10), years of providing consultation in the field of early childhood (*F* = 1.47, *p* = 0.27), gender (*X*^2^ = 0.48, *p* = 0.49), race (*X*^2^ = 1.11, *p* = 0.29), and ethnicity (*X*^2^ = 2.00, *p* = 0.37).

### 2.2 Procedures

#### 2.2.1 Virtual robotic telepresence consultation equipment and software

In the present study, we used the Kubi Plus (Xandex Inc., 2019), which is a stationary desktop telepresence robot created by Kubi Robotics that allows users to have a virtual presence in a remote location. It has a stable round base and an arm that holds a detachable tablet or iPad. The robot’s arm can pan and tilt, which gives remote users control over the camera’s field of view. This allows the remote user operating the robot to look around a room, focus on different people/objects, and follow activities, providing an opportunity for remote users to participate in meetings, consultations, and conversations more naturally compared to a stationary webcam. The Kubi (Japanese for neck) Plus stationary telepresence robot holds a tablet and can tilt +/− 45 degrees vertically and pan 360 degrees horizontally. For this study, an iPad was placed in the Kubi Plus and a powered 3.5 mm shotgun microphone and external speaker was plugged into the iPad. A shotgun microphone connected to the iPad on the Kubi Plus allowed for remote consultants to hear classroom interactions further away than a regular tablet microphone (approximately 30 feet away). Zoom Video Conferencing for Healthcare (Qumu Corporation, 2023) video conferencing software was installed on each iPad for consultation sessions. The Kubi Plus robot was connected to iPads via Bluetooth connection utilizing a Kubi Connect app installed on the iPad. Each iPad within the center had consultant Zoom (Qumu Corporation, 2023) personal meeting room links (e.g., to provide one link for meetings always instead of multiple meeting invites) and the cloud-based multilingual implementation guide links placed on the home screen to make it easier for childcare staff to connect to scheduled sessions and/or access materials on how to operate and/or troubleshoot the equipment and software. Each set of equipment was provided with a password-protected mobile hotspot to ensure a consistent internet connection that was not reliant on the childcare center’s Wi-Fi. The telepresence robot, tablet, and mobile hotspot when used together for providing virtual consultations, offers different camera fields of view so the consultant can see and hear interactions as they occur (Bice-Urbach et al., 2018; Fischer et al., 2018).

#### 2.2.2 Virtual robotic telepresence consultation training approach

**Figure 1:**
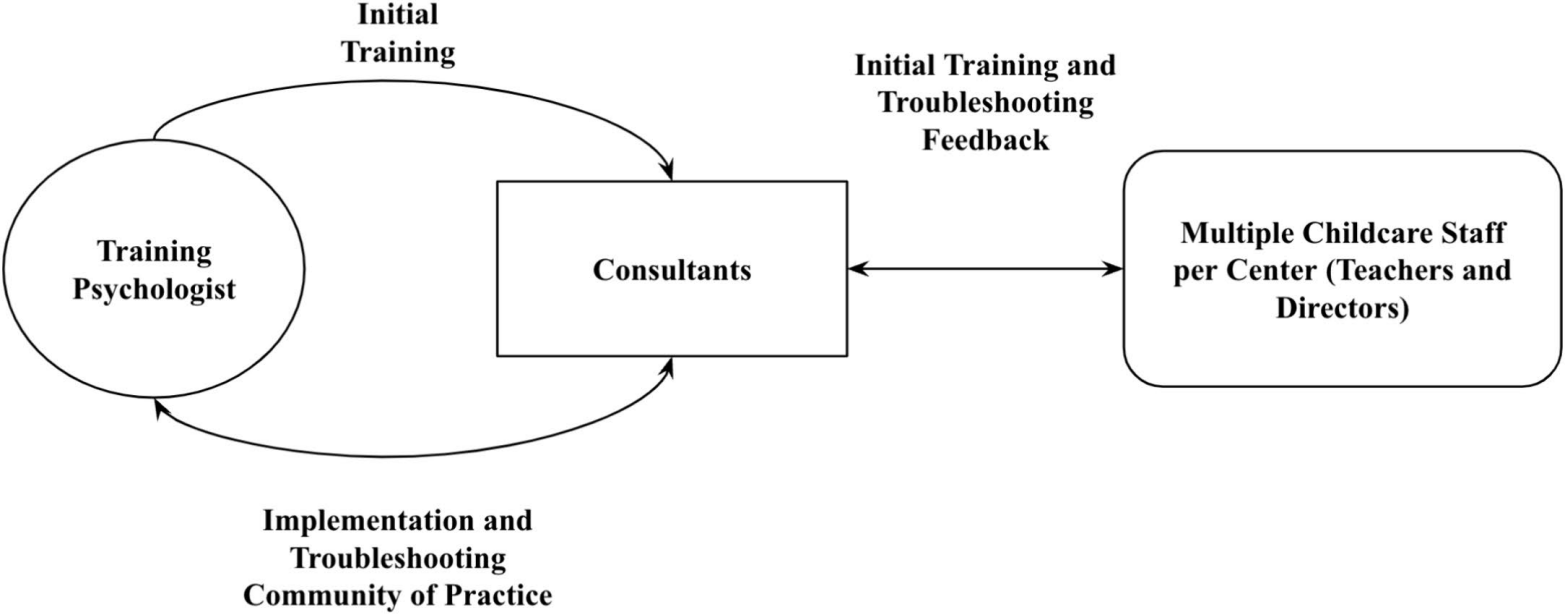
Virtual robotic telepresence consultation training approach. While initial training was provided by the psychologist, feedback from consultants and childcare staff was bidirectional, which informed implementation and troubleshooting discussions during weekly communities of practice.

Figure 1 provides an overall flow of the training, consultation, and troubleshooting process. A psychologist with expertise in technology-based interventions provided training and ongoing consultation to all consultants across both intervention groups (JS + CS and HC2) utilizing the telepresence robot. Each study consultant initially received hands-on simulation-based training (1 h) where they practiced assembling the Kubi Plus stationary telepresence robot, placing the iPad on the robot, connecting external shotgun microphones and speakers, connecting to a mobile hotspot for internet connection, and connecting the iPad to the robot via Bluetooth connection. Consultants were shown how to remotely connect to the robot and operate the pan and tilt features and then practiced operating the robot remotely until they felt comfortable with the process. Finally, consultants were shown how the placement of the robot impacted camera field of view as well as ability to hear verbalizations from further away distances. Consultants were then instructed to train the childcare staff in a similar manner to how they were trained (e.g., practicing setting up the equipment and connecting to a videoconference call) to ensure the childcare staff knew how to use the equipment and virtual consultation approach. Beyond initial training, each consultant was provided the opportunity for remote consultation from the training psychologist when setting up the robot within childcare classrooms. The training psychologist also facilitated a weekly 30-min virtual community of practice with consultants, where they could share strategies for improving implementation of the virtual robotic telepresence consultation approach (e.g., robot disconnecting from the tablet, poor internet connectivity, teachers having difficulties with remembering how to set up equipment) regardless of intervention group. During the communities of practice, some consultants expressed concern that childcare staff were experiencing difficulties with equipment or noted they needed to travel on-site to collect paper measures from the center. In those instances, consultants were instructed to deliver a hybrid session. Specifically, consultants were instructed to set up in a space outside of the classroom (e.g., typically the director’s office) and continue to connect via Zoom to deliver the virtual consultation, even though they were onsite. In these instances, consultants also had face-to-face interactions with childcare staff (e.g., troubleshooting equipment). Following the initial 30-min virtual community of practice each week, each intervention group (HC2 and JS + CS) used separate break out rooms to discuss consultation model-specific implementation. During the Cohort 1 study period (November 2022 and September 2023), 46 Communities of Practice were facilitated, and all consultants attended a minimum of 80% of all sessions offered.

Beyond live supports, a cloud-based multilingual (English and Spanish) implementation guide was developed for consultants and childcare staff to provide guidance on utilizing the virtual telepresence robotic consultation equipment and software (i.e., Kubi Plus robot, external microphone and speaker, iPad, mobile hotspot, and Zoom video conferencing software) and how to troubleshoot common issues (e.g., robot and video conferencing connectivity issues, low battery). In addition, a video-based instructional guide was developed to show consultants each step of the connection and operation process for the virtual telepresence robotic consultation approach to utilize as an on-demand resource.

Within each childcare center, consultants met with childcare staff to determine the location in the classroom for the equipment to be set up to provide the best camera field of view of childcare staff-child interactions and audio of staff-child verbalizations based on their activities throughout the day. The equipment, when possible, was placed up and away in classrooms to provide a broader camera field of view and to minimize the extent that children could reach equipment (e.g., placing electrical cords behind shelves, placing equipment on top of higher shelving within the classroom). To ensure the Kubi Plus was placed in the same mutually agreed upon location each time, stickers were placed on the spot within the classroom where the robot was to be set up. Consultants instructed teachers to keep all equipment (i.e., Kubi Plus telepresence robot, external microphone and speaker, iPad, and mobile hotspot) plugged in and together when in classrooms to reduce battery issues at the time of study consultation sessions.

Consultants provided in-person training to teachers on how to connect to the Kubi robot and Zoom video conferencing sessions using hands-on simulated approaches. They simulated initial consultation sessions by joining Zoom sessions from another room within the childcare center to ensure childcare staff knew how to operate the equipment and software prior to their first scheduled remote virtual robotic consultation session. When conducting check-ins or observations during Zoom sessions, consultants kept their camera turned off and microphone muted to minimize disrupting the classroom environment.

Consultants provided childcare staff troubleshooting when equipment and/or software did not work for them through multiple methods when on-demand materials were not enough to solve problems. Initially, consultants attempted to offer remote consultation to childcare staff via a phone call or via a Zoom video conferencing session where teachers switched to a front facing camera field of view so they could show the consultant what was not working with the Kubi. The training psychologist was also available for remote troubleshooting upon consultant request. If consultants and childcare staff could not solve problems with the virtual robotic telepresence consultation approach remotely, consultants would travel to childcare centers to problem-solve with childcare staff in-person.

#### 2.2.3 Consultant cluster random assignment

Childcare centers were randomly assigned to receive one of two interventions, JS + CS or HC2. One consultant was assigned to each childcare center and consultants only provided one type of intervention, JS + CS or HC2, across any childcare centers they were assigned. Consultants were instructed to deliver the models via a virtual robotic telepresence consultation approach. Control childcare centers received the same pre-post measures and incentives as the intervention arm to promote study retention.

##### 2.2.3.1 JumpStart + COVID support intervention

JS + CS is an early childhood mental health consultation program modeled after the evidence-based Georgetown University’s Infant/Early Childhood Mental Health Consultation (I/ECMHC) model comprised of four pillars; (1) Safety, (2) Behavior Support, (3) Self-Care, and (4) Communication. The JS + CS program is based on Caring for our Children-National-Health and Safety Standards (American Academy of Pediatrics et al., 2019) supplemented with CDC COVID-19 guidelines for childcare centers (Centers for Disease Control and Prevention, 2023), and evidence-based practices for building children’s social competence (e.g., Hemmeter et al., 2022). The JS + CS consultation model was scheduled with childcare centers for 1 h per week across 14 weeks and was provided via consultants using telepresence robots. The purpose of the JS + CS model is to: (1) improve children’s social, emotional, and behavioral development and (2) prevent and/or eliminate behavioral challenges in children by increasing the capacity all caregivers (directors, teachers, and parents/families) to address them in an effort to reduce and prevent preschool expulsions and suspensions.

Within each pillar of the JS + CS model there are a series of 24 infographics, six for each pillar, customized for either the center director or classroom teacher. Each infographic has standardized formatting consisting of three sections; (1) Reflect, (2) Inform, and (3) Practice. In addition, each infographic provides hyperlinks to online resources that childcare staff can use to support the uptake of a new skill. The infographics are PDF documents so they can be shared with program directors and teachers ahead of and during consultations via screen sharing within videoconferencing software. The infographics are a guide to lead the consultant and childcare staff through the consultative process allowing childcare staff to reflect on their own practices and jointly develop individualized professional goals. In addition to the 24 infographics, all staff receive toolkits made up of many different materials that align with each pillar. Examples of materials include disposable face masks and a no contact infrared thermometer (Safety pillar), emotion tiles and a liquid motion timer (Behavior support pillar), stress ball and a relaxation fountain (Self-care pillar), and a note to parents template and suggestion box (Communication pillar).

JS + CS consultants were clinically trained and supervised early childhood mental health consultants, many of whom are endorsed by a state association of infant mental health. Each consultant received extensive training on implementing the JS + CS program. The JS + CS consultants received a series of six virtual training sessions as part of their initial onboarding: (1) a general introduction to Georgetown University’s I/ECMHC/Jump Start model and its four program pillars (2) program-level observation training, (3) classroom-level observation training, (4) review of curriculum infographics, and (5) watched and debriefed identified exemplar program and classroom level consultation videos. Before starting baseline data collection, the two licensed lead mental health consultants (MHCs) provided hands-on training support by shadowing simulated virtual consultations.

##### 2.2.3.2 Healthy caregivers-healthy children intervention

Centers randomized to the attention control received an existing obesity prevention program, HC2 (Messiah et al., 2017; Natale et al., 2021) by consultants. HC2 consultants were trained by a doctoral level staff member with expertise in child nutrition and exercise behaviors. This program has been found to have a wide evidence base for improving obesity prevention practices within centers (Natale et al., 2014, 2017) that were ethnically similar to this study. The HC2 toolkit was implemented at the same level of exposure (14 weeks) and contact time (minimum 1 h per week) as the intervention group and was provided via consultants using telepresence robots. The same virtual multi-level system (center, classroom, and child level) mirrored the intervention group except that the four toolkit pillars were different (Screen Time, Beverage, Snack, and Physical Activity). The HC2 toolkit was designed to integrate nutrition and physical activity policies into the Quality Counts childcare center standards in Florida. The key policies that form the foundation of the HC2 toolkit include: (1) Snack Policy: favoring fresh fruits, vegetables and whole grains over sweets and high-fat foods, (2) Beverage Policy: Serving low-fat or non-fat milk, limiting juice, and encouraging water consumption, (3) Physical Activity Policy: Providing at least 90 min of physical activity daily, and (4) Screen Time Policy: Limiting screen time to less than 30 min per week. Materials consisted of written lesson plans (per pillar) to be used in the classroom; lesson plans included the lesson objective, lesson preparation, props, instructions, activities, language/vocabulary, and culturally and linguistically appropriate services (CLAS) associations and enrichment questions. The lesson plans were intended to be shared electronically, along with appropriate props that went along with each pillar/lesson plan (e.g., puppets, soccer balls, and parachutes).

HC2 consultants were trained by a licensed mental health counselor and a Doctorate-level Exercise Physiologist through two virtual training sessions that consisted of reviewing the four policies of HC2, associated classroom and program level activities, and role-playing implementation of the curriculum (Natale et al., 2014).

### 2.3 Measures

#### 2.3.1 Quantitative measures

The Technology Acceptance Model Instrument-Fast Form (FF-TAM; Chin et al., 2008) is a 16-item checklist that evaluates attitudes toward technology use. It can be modified by listing the technology of interest. Within this study, virtual robotic telepresence consultation was listed as the technology. The FF-TAM uses an 8-point semantic differential scale ranging from −4 (e.g., inefficient) to +4 (e.g., efficient) to rate items, allowing for a broad range of responses. The FF-TAM has three subscales designed to measure different aspects of technology acceptance including Usefulness (e.g., unhelpful vs. helpful), Ease of Use (e.g., very cumbersome vs. very useable), and Predicted Future Usage (e.g., For future consultation tasks that are totally within my control, I would probably use telepresence robots as a consultation platform). All three subscales (i.e., usefulness, ease of use, and predicted future usage) were used in this study. Consultants completed this measure at baseline (1 month after Kubi exposure) and post-intervention, whereas childcare staff only completed this measure immediately post-intervention. Internal consistency for the overall scale was strong for both childcare staff (*α* = 0.97) and consultants (*α* = 0.95).

An abbreviated version of the Technology Readiness Index 2.0 (Parasuraman and Colby, 2015) was utilized to screen consultant readiness to utilize technology. Four Likert scale items (1 = Strongly Disagree to 5 = Strongly Agree, one item from each subscale) were used to screen consultant’s attitudes including general optimism about technology, innovativeness, discomfort, and insecurity. Discomfort and insecurity responses were reverse coded and then all item scores were summed for an abbreviated technology readiness score. Internal consistency for the abbreviated 4-item scale was satisfactory (*α* = 0.64) in this sample, in that the brief scale appeared to measure its intended content (Taber, 2018). Specifically, the abbreviated Technology Readiness total score was positively correlated with the Technology Fast Form (Ease of Use: *r* = 0.61, *p* < 0.001, Usefulness: *r* = 0.59, *p* < 0.001, and Predicted Future Usage: *r* = 0.63, *p* < 0.001). It is likely that if more items would have been added, the value of alpha would have increased similar to the full-length Technology Readiness Index 2.0, in which four subscales ranged from Cronbach αs of 0.70 to 0.83 within an initial measure validation study (Parasuraman and Colby, 2015; Taber, 2018).

The percentage of virtual robotic consultations per childcare staff member was calculated by dividing the total number of virtual robotic telepresence consultations by the total number of consultation sessions received by each childcare staff member. Consultation format of delivery was documented. Sessions were conducted either in-person, via virtual robotic telepresence consultation, and/or hybrid format (e.g., part of consult was in-person and part of consult was conducted remotely via phone or virtual robotic telepresence consultation).

#### 2.3.2 Qualitative measures

Two focus groups were conducted with consultants (*N* = 10 total consultants; 100% participation) from each of the study conditions after the intervention period. During the focus groups, participants responded to a series of open-ended questions in real time using the Mentimeter polling platform (Mentimeter, 2023). See Table 2 for a list of questions. Examples of questions included, “*Tell me about your experiences with Kubi training including using the Kubi written instructional guide and demonstration video*” and “*How do you think the teachers/site directors perceived the Kubi teleconsultation approach at the end of implementation?*” The focus group facilitator (a member of the investigative team not directly involved in study implementation with expertise in qualitative methods) viewed participants’ anonymous written responses as they were submitted and prompted them to expand on their comments as needed. We recorded these discussions, which lasted approximately 1 h, via Zoom video conference. Participants’ responses to open-ended questions were recorded by the Mentimeter software, and Zoom produced verbatim transcripts of the discussion.

**Table 2:** Focus group interview guide.

1. What percent of the time do you use the Kubi for the following activities: Consultations? Observations? 2. Tell me about your experience using the Kubi. 3. Tell me about your experiences with Kubi training including using the Kubi written instructional guide and demonstration video. 4. What, if anything, would improve the Kubi training you received, knowing what you know now? 5. What made it easy to train the school personnel to use the Kubi? 6. What made it difficult to train the school personnel to use the Kubi? 7. Describe the most common/frequent technological issues that arose when you were attempting Kubi teleconsultation meetings. 8. What could have helped to prevent these technological issues from happening? 9. If/when you ran into technology issues, what approach did you take to troubleshoot? 10. How, if at all, do you feel like the Kubi teleconsultation approach worked for providing services in childcare centers? 11. How do you think the teachers/site directors perceived the Kubi teleconsultation approach at the beginning of services? 12. How do you think the teachers/site directors perceived the Kubi teleconsultation approach at the end of implementation? 13. What, if any, technological features would have made the Kubi more useful (e.g., longer battery charge)? 14. What, if anything, would make it more likely for you to use Kubi teleconsultation in childcare centers in the future? 15. What, if anything, would make teachers/site directors more likely to use Kubi teleconsultation in childcare centers in the future? 16. Thinking through technology used in this project and how we delivered the services, what would you change in our delivery of Jump Start+/HC2?

### 2.4 Data analytic approach

Descriptive analyses were conducted for measures completed by consultants and childcare staff. Before proceeding with paired sample t-tests, one-way ANOVAs were conducted to see if there were any differences between the intervention and attention control consultants in baseline perceptions of technology acceptability. There were no significant differences between consultant groups (JS + CS and HC2) on baseline perceptions of usefulness (*F* = 0.01, *p* = 0.93), ease of use (*F* = 1.52, *p* = 0.25), or predicted future usage (*F* = 0.59, *p* = 0.46). Therefore, consultant groups were then combined to examine any baseline-post changes in consultant perceptions of the usefulness, ease of use, and predicted future use of the virtual telepresence robotic consultation approach, given the small sample size of consultants. Paired sample t-tests were conducted to examine baseline-post changes in consultants’ perceptions.

Given that the intervention was designed to be delivered exclusively virtually, but consultants did not consistently implement consultations within this format, differences in consultation model fidelity (i.e., all consultations delivered virtually) was examined. Chi-Square analyses (*Χ*^2^ = 13.91, *p* < 0.001) revealed that only a portion of teachers in the JS + CS intervention group received 100% of their consultations virtually and none of the teachers within the HC2 group received 100% of their consultations virtually. Therefore, to control for the potential confounding effects for teachers who received consultation completely virtually and those who did not, intervention group was listed as a fixed effect within all path analyses.

The data was analyzed following recommended procedures (McDonald, 2014; Kline, 2023). Next, we examined whether teacher, consultant, and childcare center variables predicted virtual robotic telepresence consultation uptake and childcare staff technology outcomes. In alignment with best practices (Kline, 2023), these analyses proceeded in a series of several steps. First, data were nested by both childcare center and consultant. Therefore, for each outcome variable, a preliminary 3-level unconditional random effects ANOVA to determine whether significant variance in each outcome variable was accounted for at the consultant and childcare center levels. We did so by calculating intraclass correlations (ICCs) at each level. A statistically significant ICC indicates that significant amounts of variance in an outcome are attributable to differences between consultants and/or childcare center, and that such nesting effects should be accounted for in subsequent modeling. As reported below in the results, ICCs were not statistically significant in any of the empty models at the consultant level. Therefore, in subsequent models, we included consultant as a covariate to control for consultant effects, but only accounted for nesting at the childcare center level (2-level model).

Second, after investigating unconditional random effect ANOVAs, we used path analysis in Mplus to examine whether teacher, consultant, and childcare-level variables predicted virtual robotic telepresence consultation uptake and childcare staff technology outcomes. These analyses were conducted using full information maximum likelihood robust estimation procedures (MLR) in Mplus. These estimation procedures ensured all responses, even those with partially missing data, were included in study analyses (Kline, 2023). The use of such estimation procedures ensures that an intention-to-treat framework was used in all analyses that included all teachers and consultants who began the study, even if they did not complete the intervention. Using an intention-to-treat approach ensures outcome estimates are not biased by only including participants who completed the intervention (Rothenberg et al., 2020). To control for clustering of teachers within childcare centers, the “Cluster=” command was used, and childcare center was included as the clustering variable. All models demonstrated good fit per recommended cutoffs (CFI/TLI ≥ 0.95, SRMR ≤0.08, RMSEA ≤0.10; Kline, 2023).

The path analysis predicting percentage of virtual consultations included teacher predictors (i.e., childcare staff age, primary language), school predictors (i.e., JS + CS or HC2 intervention assignment), and consultant predictors (i.e., baseline perceptions of the usefulness, the ease of use of the virtual robotic telepresence consultation approach, and consultant general perceptions of technology readiness).

The path analysis predicting childcare staff post intervention perceptions of virtual telepresence robotic consultation usefulness included teacher (i.e., childcare staff age, primary language), school (i.e., JS + CS or HC2 intervention assignment), and consultant predictors (i.e., baseline perceptions of the usefulness of the virtual telepresence consultation approach and consultant general perceptions of technology readiness).

The path analysis predicting childcare staff post intervention perceptions of virtual telepresence robotic consultation ease of use included teacher (i.e., childcare staff age, primary language), school (i.e., JS + CS or HC2 intervention assignment), and consultant predictors (i.e., consultants baseline perceptions of the ease of use of the virtual telepresence consultation approach and general perceptions of technology readiness).

Finally, the path analysis predicting childcare staff post intervention perceptions of virtual telepresence robotic consultation predicted future usage included teacher (i.e., childcare staff age, primary language), school (i.e., JS + CS or HC2 intervention assignment), and consultant predictors (i.e., consultants baseline perceptions of the predicted future usage of the virtual robotic telepresence consultation approach and general perceptions of technology readiness).

Rapid qualitative analysis (RQA; Hamilton, 2013, 2020; Hamilton and Finley, 2019) was used to summarize key points and explore relevant themes from consultant focus groups. The team followed a systematic process that involved two analysts reviewing consultants’ Mentimeter poll responses and Zoom transcripts. The first analyst completed the initial interview summary using a standard template that summarizes key points for each question. The second team member then audited the summary template for accuracy and transferred key statements into a matrix that contained all participant responses for each Mentimeter question. Co-authors used the matrix to generate preliminary themes, which were discussed and refined by the larger research team.

## 3 Results

Pre-post paired sample *t*-tests were conducted to determine if consultant perceptions of the usefulness, ease of use, and predicted future usage of the virtual robotic telepresence consultation approach changed over time. Despite initial training and ongoing weekly communities of practice, consultant perceptions of the virtual robotic telepresence consultation approach did not change over time (see Table 3). Consultants had a generally positive view of the virtual robotic telepresence consultation usefulness (Post *M* = 2.12, *SD* = 1.23) and ease of use (*M* = 2.05, *SD* = 1.52) but were less positive about predicted future usage of the consultation approach (*M* = 0.86, *SD* = 2.06). Descriptively, childcare center directors and teachers had slightly more positive views of the virtual robotic telepresence consultation usefulness (Post *M* = 2.99, *SD* = 1.44), ease of use (*M* = 2.95, *SD* = 1.43), and predicted future usage (*M* = 2.60, *SD* = 1.62) than consultants. Despite the interventions being designed to be exclusively delivered via virtual telepresence robotic consultation, an average of 46% of consultations per childcare staff were conducted virtually, 44% of consultations were conducted in-person, and 10% of consultations were conducted in a hybrid format. Of note, consultants did not disclose the extent that consultations were being conducted in-person during the study period communities of practice.

**Table 3:** Paired sample *t*-test results for Technology Acceptance Model Instrument-Fast Form.

Scale	*N*	Pre *M*	*SD*	Post *M*	*SD*	*t*
Usefulness	7	2.36	1.27	2.12	1.23	0.44
Ease of use	7	2.43	1.78	2.05	1.52	0.86
Predicted usage	9	1.69	1.78	0.86	2.06	0.96

No significant pre-post differences.

Unconditional random effects ANOVAs showed 32% of variance in virtual consultation percentage scores was due to differences between childcare centers (intraclass correlation [ICC] = 0.32, *p* = 0.04), and 56% was due to differences between staff assigned to consultants (ICC = 0.56, *p* = 0.09). For usefulness perceptions, 24% of variance was attributable to childcare center differences (ICC = 0.24, *p* = 0.09), and 23% to consultant differences (ICC = 0.23, *p* = 0.17). For ease of use perceptions, 54% of variance was attributable to childcare centers (ICC = 0.54, *p* = 0.03), and 16% to consultants (ICC = 0.16, *p* = 0.28). For predicted future use, 24% of variance was due to childcare centers (ICC = 0.24, *p* = 0.11), and 19% to consultants (ICC = 0.19, *p* = 0.19). Across all four models, only childcare center variance estimates were significant at *p* < 0.05 (consultant variance estimates were never significant), therefore, it was determined appropriate to control for nesting of staff within childcare centers in all analyses (Bauer and Curran, 2021).

Path analyses were conducted to determine predictors of virtual robotic telepresence consultation completion rates. Older childcare staff (*B* = 0.007, *SE* = 0.003, *t* = 2.524, *p* = 0.012) and childcare staff assigned to the JS + CS intervention group (*B* = 0.506, *SE* = 0.163, *t* = 3.114, *p* = 0.002) were more likely to receive a higher percentage of services via virtual robotic telepresence consultations compared to younger childcare staff (see Table 4). Childcare staff language and consultant technology readiness and perceptions of acceptability of the virtual robotic telepresence consultation approach did not impact childcare staff uptake of the virtual approach.

**Table 4:** Impact of teacher, childcare center, and consultant characteristics on virtual telepresence robotic consultation completion.

Fixed Effect	*B*	*SE*	*t*	*p*
Consultant	−0.006	0.031	−0.188	0.851
Childcare staff age	0.007	0.003	2.524	0.012
Childcare staff language	−0.094	0.077	−1.221	0.222
Intervention group	0.506	0.163	3.114	0.002
Consultant technology readiness	−0.018	0.041	−0.444	0.657
Consultant perceptions of usefulness of virtual telepresence consultation approach	0.05	0.152	0.332	0.74
Consultant perceptions of ease of use of virtual telepresence consultation approach	0.006	0.085	0.073	0.942

*N* = 72. Dependent variable: percentage of Total Consultations that were completed via the telepresence robotic consultation approach.

Path analyses were also conducted to examine the impact of various teacher, childcare staff, and consultant characteristics on post-intervention childcare staff perceptions of the usefulness of a virtual telepresence robotic consultation approach (see Table 5). Results indicated that consultants’ technology readiness (*B* = 0.611, *SE* = 0.265, *t* = 2.300, *p* = 0.021) and their baseline perceptions of the usefulness of the robotic virtual telepresence consultation approach (*B* = −0.856, *SE* = 0.315, *t* = −2.717, *p* = 0.007) were significant predictors of post-intervention childcare staff perceptions. Specifically, higher levels of consultant optimism and comfort with using technology were associated with more positive post-intervention perceptions of the usefulness of the virtual telepresence robotic consultation approach among childcare staff. Childcare staff age and language and intervention group assignment did not significantly impact childcare staff perceptions of the usefulness of a virtual telepresence robotic consultation approach.

**Table 5:** Impact of teacher, childcare center, and consultant characteristics on post intervention childcare staff perceptions of the usefulness of a virtual telepresence robotic consultation approach.

Fixed effect	*B*	*SE*	*t*	*p*
Consultant	−0.043	0.069	−0.615	0.538
Childcare staff age	0.022	0.014	1.601	0.109
Childcare staff language	0.773	0.453	1.705	0.088
Intervention group	−0.673	0.807	−0.834	0.404
Consultant technology readiness	0.611	0.265	2.300	0.021
Baseline consultant perceptions of usefulness of robotic virtual telepresence consultation approach	−0.856	0.315	−2.717	0.007

*N* = 72. Dependent variable: post childcare staff perception of the usefulness of the telepresence robotic consultation approach.

Path analyses were conducted to determine predictors of various teacher, childcare staff, and consultant characteristics on post-intervention childcare staff perceptions of the ease of use of the virtual telepresence robotic consultation approach. No significant predictors emerged within this model (see Table 6).

**Table 6:** Impact of teacher, childcare center, and consultant characteristics on post intervention childcare staff perceptions of the ease of use of a virtual telepresence robotic consultation approach.

Fixed effect	*B*	*SE*	*t*	*p*
Consultant	0.039	0.097	0.401	0.688
Childcare staff age	0.028	0.017	1.638	0.101
Childcare staff language	0.919	0.539	1.704	0.088
Intervention group	0.278	0.791	0.352	0.725
Consultant technology readiness	0.294	0.274	1.071	0.284
Pre consultant perceptions of ease of use robotic virtual telepresence consultation approach	−0.144	0.271	−0.531	0.596

*N* = 72. Dependent variable: post childcare staff perception of the ease of use of the telepresence robotic consultation approach.

Path analyses were also completed to examine various teacher, childcare staff, and consultant characteristics on post-intervention childcare staff perceptions of their predicted future usage of a virtual telepresence robotic consultation approach within childcare centers (see Table 7). The results indicate that two variables significantly predicted post-intervention childcare staff perceptions of the predicted future usage of the virtual telepresence robotic consultation approach: childcare staff age (*B* = 0.037, *SE* = 0.015, *t* = 2.461, *p* = 0.014) and consultant technology readiness (*B* = 0.629, *SE* = 0.319, *t* = 1.972, *p* = 0.049). Specifically, older childcare staff members were more likely to have more positive perceptions of future usage of a virtual telepresence robotic consultation approach compared to younger childcare staff. In addition, higher levels of consultant optimism and comfort with using technology were associated with more positive ratings of the likely future usage of the virtual telepresence robotic consultation approach by childcare staff. Childcare staff language, intervention group assignment, and consultant perceptions of predicted future usage did not significantly predict childcare staff perceptions of predicted future usage of the virtual robotic consultation approach.

**Table 7:** Impact of teacher, childcare center, and consultant characteristics on childcare staff perceptions of the predicted future usage of a virtual telepresence robotic consultation approach.

Fixed Effect	*B*	*SE*	*t*	*p*
Consultant	−0.071	0.079	−0.901	0.367
Childcare staff age	0.037	0.015	2.461	0.014
Childcare staff language	0.309	0.418	0.738	0.460
Intervention group	−1.09	0.884	−1.233	0.218
Consultant technology readiness	0.629	0.319	1.972	0.049
Pre consultant perceptions of predicted future usage of robotic virtual telepresence consultation approach	−0.394	0.238	−1.656	0.098

*N* = 72. Dependent variable: post childcare staff perception of predicted future usage of the telepresence robotic consultation approach.

### 3.1 Rapid qualitative analysis of consultant focus groups

We generated four themes related to consultants’ experiences and feedback with the Kubi Plus stationary telepresence robots for conducting classroom observations and delivering telehealth consultations in early childcare centers: (1) use practice drills to improve consultant training, (2) anticipate technological issues and treat training as an ongoing process, (3) acknowledge the benefits of teleconsultation approaches, and (4) be responsive to center preferences and consider hybrid approaches. We summarized these themes and provided corresponding illustrative quotes from consultants below.

#### 3.1.1 Theme 1: use practice drills to improve consultant training

Consultants described training materials, including the written instructional guide and demonstration video, as “helpful,” “very detailed,” and “informative.” However, they expressed a desire for additional “practice drills,” or hands-on simulation exercises as a standard part of initial training. For example, one JS + CS consultant noted, “Even though the instructional guide is helpful, what I found most helpful was the in-person training that was provided … since we got to practice how to use the Kubi.” Similarly, another JS + CS consultant shared, “[it was] more useful to use it in person over the video demonstration.” Consultants found it highly challenging to take on the role of trainers themselves to teach childcare center staff to use the Kubis. They cited “personnel’s lack of experience with technology,” and “[working with] staff members who were not as open with technology” as barriers to training school personnel. One JS + CS consultant noted, “It’s difficult (not impossible and requires patience) to train participants that are not tech-savvy. Some directors and teachers were resistant to technology since they prefer in-person consultations.” Another HC2 consultant echoed this sentiment, noting that just as with consultants themselves, opportunities for rehearsal and continued use were beneficial: “Teachers and center directors were not really too good with technology, so it made it more of a challenge at first, but over routine of using the Kubi, it got easier.”

#### 3.1.2 Theme 2: anticipate technological issues and treat training as an ongoing process

Consultants reported that the most common technological issues were related to the equipment not being adequately charged and connectivity issues (Internet and cables). To overcome these technological issues, consultants recommended conceptualizing the process of training school personnel as ongoing rather than a single meeting prior to implementation (e.g., use of “booster trainings when issues are frequent”) and noted the importance of having patience and using “frequent reminders prior to and on the day of to double check everything is ready for the observation,” to prevent technological issues. Consultants also recommended strategies such as “having iPads updated/checked after each year of use” and “checking microphone, batteries, [and] software updates.”

#### 3.1.3 Theme 3: acknowledge the benefits of teleconsultation approaches

Consultants acknowledged the utility and convenience of the Kubi, with statements such as “[it] allowed for ease of communication,” and “[it worked] really well, especially for observations [and] allowed both the consultant and directors/teachers flexibility in their schedules.” Consultants reported feeling center staff were initially resistant to Kubi uptake, expressing they were “guarded … [and] pushing for in-person,” “overwhelmed by it,” “hated it because they are non-tech,” or “intimidated to be working with new, expensive technologies.” However, they noted that toward the end of implementation, perceptions of center staff changed. HC2 consultants noted, “they loved it and liked that it was less disruptive to the classroom compared to alternatives,” and “they loved it because it made their work easier and they saw it as more convenient.” JS + CS consultants shared similar thoughts, emphasizing that “benefits, access to support” and “accessibility” to the consultant would likely make school personnel more likely to use Kubi teleconsultation in childcare centers in the future.

#### 3.1.4 Theme 4: be responsive to center preferences and consider hybrid approaches

Consultants discussed the importance of being flexible with childcare sites regarding virtual vs. in-person intervention delivery, highlighting the importance of striking a balance between center preferences and the status of a potential public health emergency in determining the appropriate modality. A JS + CS consultant shared:

“But for those centers that maybe were a lot more hesitant or had just a lot more difficulties with the tech part or who just genuinely wanted more in person connections… Also, this is like post main pandemic of COVID, but I think having that flexibility in these times [is important].”

Other JS + CS consultants shared: “It depends on the site. One site embraced Kubis and thought it was great, another site still would prefer in-person services,” and “maybe they are going to be a little bit more accepting of technology if we phrase it as hybrid versus you are diving into technology all the way.” Another consultant commented that after a childcare staff canceled multiple consultation sessions, the consultant showed up in-person to complete the intervention and found this strategy to be helpful with retaining the staff member in the intervention.

## 4 Discussion

The purpose of this study was to evaluate how training and ongoing communities of practice for consultants and childcare staff impacted uptake and acceptability of a virtual telepresence robotic consultation approach for providing early childhood mental health consultation services. Overall, the results provide some support for the acceptability of utilizing stationary telepresence robots to provide virtual consultation services within childcare centers when proper training and ongoing support is provided. It is important to note this approach was implemented in predominantly low-income Hispanic communities where the majority of childcare staff’s primary language was Spanish. Historically, there have been disparities in technology uptake within low income and historically marginalized communities (Porter and Donthu, 2006; Katz and Gonzalez, 2016; Anderson-Lewis et al., 2018). This study provides some support for increasing technological reach within this important underserved community of childcare staff, but there were barriers to implementation and adoption of this technology.

### 4.1 Implementation

Fidelity to the intended model was limited. Despite virtual telepresence robotic consultation being the intended modality, only 46% of consultation sessions per childcare staff on average were conducted fully virtually. The extent consultation sessions were conducted via in-person or hybrid formats was not disclosed by consultants during ongoing communities of practice, which limited the extent barriers could have been problem-solved during weekly meetings. While behavior observations of fidelity are administered at baseline and at post-intervention as a part of the larger trial, ongoing behavior observations were not conducted during the intervention which would have likely revealed the relatively low virtual consultation rate. Focus groups with consultants elucidated there was variability in childcare staff preferences for virtual or in-person consultation and expressed that childcare staff and the consultants themselves had an increased desire for providing in-person consultation once COVID-19 restrictions were lifted. The shift to virtual consultations during COVID-19, while necessary for safety reasons, introduced new obstacles such as technological issues, scheduling conflicts, and competing priorities for both consultants and childcare staff. As a result, consultants may have needed to balance retaining childcare staff versus mandating that consultations be conducted virtually, even if it meant being flexible with scheduling or adapting the delivery format. This determination by consultants to support childcare staff to implement the interventions based on childcare staff preferences, highlights the resilience required to navigate the unforeseen complexities of providing services during a global pandemic.

Consultants also expressed that childcare staff were sometimes resistant initially to rely exclusively on virtual services. Over time, childcare staff acknowledged the convenience of the virtual robotic telepresence consultation approach once comfortable with using the technology, as found in previous research where new technology was introduced within educational settings (Schultz et al., 2017). This finding highlights an ongoing need for childcare staff to receive hands-on training and support throughout the intervention periods as they learn to adopt a new technology within classroom settings.

Interestingly, older childcare staff were more likely to receive a higher percentage of consultations virtually. While we did not examine childcare staff years of experience directly, these results suggest, by proxy, it is possible that older teachers had more years of experience within childcare settings, which may serve as a facilitator regarding implementation. That is, it is possible that with more years of experience, older childcare staff may have a stronger sense of teaching efficacy. This efficacy may allow them to be more open to trying new approaches, such as a virtual telepresence robotic consultation approach, as they may feel more equipped to adapt and integrate new approaches like virtual robotic telepresence consultation into their existing teaching practices. However, these findings are inconsistent with prior examinations of teachers’ use of technology within classrooms, where teachers with more experience were more likely to be categorized as low technology users within their classrooms with preschool children (Dore and Dynia, 2020).

The intervention consultation model utilized may also influence the uptake of the virtual robotic telepresence consultation approach. The JS + CS consultation model is more discussion- and observation-based, potentially lending itself better to a virtual robotic telepresence consultation approach, whereas the HC2 curriculum relies more on modeling of physical content within the classroom and practicing skills with in-person materials (e.g., parachutes, soccer balls). This may have made it more difficult for consultants to implement the HC2 model virtually. Therefore, consultation models and the specific outcomes hoped to be gained through a virtual robotic telepresence consultation approach should be considered when determining best fit for format.

Qualitative data also provided insight as to how to improve the training and childcare center classroom implementation of the virtual robotic telepresence consultation approach. While consultants appreciated initial training efforts, they thought additional practice drills on how to set up and troubleshoot the equipment and software would have improved consultant preparedness for implementation. Trainees often learn better from experiential learning approaches than passive learning (Kolb and Kolb, 2017). Flipped classroom approaches should be considered where trainees receive didactics online in advance and training is focused on active practice of skills. Specifically, simulation practice could be established where some consultants role play as “childcare staff” that do not know how to set up equipment and consultants have to teach them. Consultants could also practice scenarios remotely in which they have to troubleshoot a variety of scenarios (e.g., robot not turning on, robot not connecting to iPad, different warning lights going off on robot, unplugged equipment) with simulated “childcare staff.”

Focus groups also noted that childcare staff training on troubleshooting the virtual robotic consultation approach should be viewed as an ongoing process requiring patience and reminders, which mirrors prior teleconsultation research (Ihorn and Arora, 2018). Some consultants recommended providing formal in-services where childcare staff could earn continuing education to potentially increase staff buy-in into learning how to use the technology and equipment. This virtual approach was new to all consultants and childcare staff so it was difficult to anticipate what specific problems would emerge with equipment and software. While technological issues were identified and discussed within communities of practice, subsequent hands-on simulated practice should have been provided to consultants to navigate troubleshooting new issues as they were identified (e.g., iPad software updated and lost Bluetooth connection to the Kubi Plus robot).

### 4.2 Adoption

Several consultant and childcare staff characteristics appear to impact adoption of the virtual telepresence consultation model. Consultants shared several barriers and facilitators of this approach. Childcare staff had moderately positive views of the usefulness, ease of use, and predicted future usage of the virtual telepresence robotic consultation approach after intervention implementation. Similarly, during focus groups, consultants reported the virtual robotic telepresence consultation approach was beneficial. However, they noted it took time and repeated practice with the equipment before both the consultants and childcare staff felt comfortable with and accepted this format of consultation. These findings align with prior research that shows educators have generally viewed teleconsultation approaches as acceptable (Bice-Urbach and Kratochwill, 2016; Fischer et al., 2016; Ihorn and Arora, 2018).

Consultants were somewhat less positive about the predicted future usage of the virtual telepresence robotic consultation approach than childcare staff. As noted in the focus groups, consultants often had to provide extra in-person support to childcare staff to implement the virtual approach initially. This extra workload may have been a barrier for consultants to view this approach as a long-term viable option for service delivery.

Consultant attitudes such as more comfort and readiness to use new technology was related to childcare staff having more positive views of the usefulness of the virtual telepresence robotic consultation approach. It is possible these consultants were more engaged and patient in helping childcare staff understand how to utilize the technology and ultimately experience positive benefits of the virtual approach. However, the extent that consultants provided additional technological assistance and troubleshooting to childcare staff was not measured.

The negative relationship between consultants’ initial perceptions of the usefulness of the virtual telepresence robotic consultation approach and childcare staff’s post-intervention perceptions is noteworthy and requires further exploration. One potential explanation is that consultants had high expectations for the virtual consultation approach and communicated these to the childcare staff. It is possible that any failures (e.g., ongoing technology or connection problems) to meet these expectations during the actual consultation experience could have led to diminished childcare staff expectations about the usefulness of the virtual consultation approach. Also, as COVID-19 restrictions decreased and some childcare staff reported an interest in a return to in-person consultation services, they may have perceived the virtual consultation approach as less useful, as having a consultant show up in their classroom requires less preparation than setting up equipment for virtual consultation.

### 4.3 Limitations and future directions

This study had multiple limitations that should be noted. The study had a relatively small sample size of childcare centers and consultants limiting generalizability. However, this study used a convergent parallel mixed methods design to understand consultant and childcare staff uptake and perceptions of the virtual robotic telepresence consultation model. Further, childcare centers were all serving predominantly Hispanic families and low-income families. Findings may not generalize to other populations. A significant limitation of the present study stems from the fact that it was designed for a larger cluster randomized controlled trial investigating virtual consultation. However, in this pilot, only 46% of the consultation sessions were actually delivered through the virtual consultation approach, with the remaining sessions being conducted in-person or via a hybrid format. Consequently, the consultants and the childcare staff’s ratings of the virtual consultation approach were based on limited exposure to the intended delivery modality in some instances. That is, consultants and childcare staff experienced fewer virtual sessions than what would have been provided had the consultants adhered to the study protocol. Further, we did not measure the number of initial virtual telepresence robotic consultation training/troubleshooting sessions that consultants offered childcare staff, which would have helped to better understand breakdowns in the technology transfer of knowledge and skills from consultants to childcare staff. Another limitation of the current study was the lack of detailed tracking of the specific content covered during the virtual consultation sessions. Without this information, it was not possible to determine which aspects of the JS + CS and HC2 consultation curriculums were more effectively implemented virtually. This insight could have provided valuable information for optimizing the virtual consultation approach and identifying areas where the curriculum may need to be adapted to increase fidelity of virtual delivery. To address this limitation, future cohorts of this study should incorporate more frequent behavioral observations of consultation sessions across interventions to gain a deeper understanding of which aspects of the curriculum are most conducive to virtual implementation.

Overall, this study provides some initial support for a virtual robotic telepresence consultation approach that allowed for service provision when traditional in-person consultation was not possible during a public health emergency. The virtual robotic telepresence model has significant implications for improving access to specialized support services while maintaining public health protections, in the event of a future pandemic. However, a hybrid consultation approach balancing virtual and in-person interactions may lead to the highest satisfaction and uptake among both consultants and childcare staff, when feasible. Training consultants and childcare staff requires initial active practice opportunities and an ongoing simulated practice. Based on the findings from the current study, virtual robotic telepresence consultation approach should include a multiphase approach to training as follows: (1) training the consultant how to effectively utilize the technology, (2) training the consultant on how to train educational staff to utilize the technology, (3) providing ongoing simulated practice of troubleshooting support to consultants and childcare centers to promote long-term sustainability, and (4) provide ongoing behavior observations of virtual delivery of services early and often to better identify issues related to fidelity and adoption. Embedded technology support specialists such as childcare center directors within these settings who develop expertise in utilizing new technologies may support sustainability of the virtual robotic telepresence consultation approach in centers, where there are high staff turnover rates (NSECE, 2013).

Future research trials should examine childcare-centered decision-making regarding the format of consultations (i.e., virtual, in-person, and hybrid) to allow for optimal personalization of the consultation approach to person and setting. Research on virtual telepresence robotic consultation training approaches that go beyond initial adoption and implementation are warranted to determine facilitators and barriers to hybrid consultation models as well as sustainability of technology use within educational settings. Lessons learned can inform how best to utilize virtual robotic telepresence consultation approaches to increase reach of services well beyond the childcare setting.

## Data availability statement

The raw data supporting the conclusions of this article will be made available by the authors, without undue reservation.

## Ethics statement

The studies involving humans were approved by University of Miami Institutional Review Board. The studies were conducted in accordance with the local legislation and institutional requirements. The participants provided their written informed consent to participate in this study.

## Author contributions

JJ: Conceptualization, Data curation, Formal analysis, Investigation, Methodology, Supervision, Writing – original draft, Writing – review & editing. SS: Conceptualization, Data curation, Formal analysis, Investigation, Methodology, Writing – original draft, Writing – review & editing. YA: Data curation, Formal analysis, Methodology, Project administration, Writing – original draft. WR: Formal analysis, Writing – original draft, Writing – review & editing. EH: Methodology, Project administration, Writing – original draft, Writing – review & editing. CV: Project administration, Writing – original draft. EM: Writing – review & editing. EG: Investigation, Writing – review & editing. RB-S: Conceptualization, Writing – review & editing. RN: Conceptualization, Investigation, Methodology, Supervision, Writing – original draft, Writing – review & editing.
